# A simplified and highly efficient cell-free protein synthesis system for prokaryotes

**DOI:** 10.7554/eLife.109495

**Published:** 2026-09-08

**Authors:** Xianshengjie Lang, Changbin Zhang, Jingxuan Lin, Zhe Zhang, Wenfei Li

**Affiliations:** 1 School of Basic Medical Sciences, Cheeloo College of Medicine, Shandong University Jinan China; 2 Children's Hospital Affiliated to Shandong University Jinan China; Murang'a University of Technology Kenya; University of Cambridge United Kingdom

**Keywords:** cell-free protein synthesis, *Escherichia coli *extract, protein production, synthetic biology, *E. coli*

## Abstract

Cell-free protein synthesis (CFPS) systems are a powerful platform with immense potential in fundamental research, biotechnology, and synthetic biology. Conventional prokaryotic CFPS systems, particularly those derived from *Escherichia coli*, often rely on complex reaction buffers containing up to 35 components, limiting their widespread adoption and systematic optimization. Here, we present an optimized *E. coli* cell-free protein synthesis (*e*CFPS) system, which is significantly streamlined for high efficiency. Through systematic screening, we successfully reduced the essential core reaction components from 35 to a core set of 7. The thorough optimization of these seven key components ensured that protein expression levels were not only maintained but even substantially improved. Furthermore, we developed a much simpler procedure for preparing the bacterial cytosolic extracts, a ‘fast lysate’ protocol that eliminates the traditional time-consuming runoff and dialysis steps, thereby enhancing the overall accessibility and robustness of *e*CFPS. This optimized and user-friendly *e*CFPS efficiently synthesizes challenging proteins, including functional, self-assembling vimentin, and active restriction endonuclease *Bsa*I despite its strong cytotoxicity, and serves as a powerful tool that will facilitate diverse applications in basic life science research and beyond.

## Introduction

Cell-free protein synthesis (CFPS) offers a powerful and flexible platform for biological research and biotechnological applications by reproducing the cellular protein synthesis process in an in vitro environment (Carlson et al., 2012; Katzen et al., 2005). Compared to traditional cell-based expression systems, CFPS possesses several notable advantages, including rapid reaction kinetics, ease of operation, independence from cell culture, high tolerance to toxic proteins, and facile incorporation of non-canonical amino acids (Martin et al., 2018; Liu and Schultz, 2010; Dumas et al., 2015). These characteristics position CFPS for broad applications in protein engineering, high-throughput screening, synthetic biology, and diagnostic reagent development (Guzman-Chavez et al., 2022; Gregorio et al., 2019).

Prokaryotic CFPS systems, especially those based on *Escherichia coli* lysates, have garnered significant attention for their high protein synthesis yields and compatibility with genetic manipulations (Jiang et al., 2021; Xu et al., 2020; Zemella et al., 2015; Jiang et al., 2018; Endoh et al., 2006; Ji et al., 2025). They have played a pivotal role in early molecular biology research and continue to be relevant in industrial protein production and biosensor development (Carlson et al., 2012; Gao et al., 2018; Lee and Kim, 2018; Meng et al., 2023). However, for a long time, most prokaryotic CFPS protocols have relied on complex buffer systems containing a large number of auxiliary components (Kigawa et al., 1999; Kim et al., 2020). A comprehensive analysis of existing literature reveals that the composition of reaction buffers differs widely between protocols, both in the number of optional components and in the concentrations of individual components (Supplementary file 1). These complex systems, while effective, often lead to high costs, laborious preparation procedures, and potential interactions among components, posing significant challenges for further optimization and standardization, thereby limiting their widespread adoption in resource-constrained laboratories.

In recent years, the field of eukaryotic CFPS has seen remarkable progress in system optimization and simplification (Bothe and Ban, 2024; Aleksashin et al., 2023). Highly optimized human in vitro translation systems have demonstrated that high-efficiency protein synthesis can be achieved with a minimal number of core components (Bothe and Ban, 2024). Recognizing the potential of such systematic optimization approaches, we hypothesized that similar strategies could be equally applicable and beneficial for *E. coli* CFPS systems by thoroughly analyzing the functions of existing components and integrating insights from previous reports on prokaryotic protein synthesis mechanisms. This study aimed to develop a highly simplified yet efficient *E. coli* cell-free protein synthesis system (*e*CFPS). We conducted a systematic component reduction screening of traditional *e*CFPS systems, successfully streamlining the core components from 35 to just 7. The subsequent meticulous optimization of these seven key components not only maintained but even improved protein expression levels. Through these dual efforts—simplifying the reaction mixture and developing a high-quality, dialysis-free ‘fast lysate’ preparation—we established a highly accessible and robust *e*CFPS platform which will accelerate diverse applications in life science research.

## Results

### Streamlining *e*CFPS: Removal of dispensable components

To develop a more streamlined and efficient *e*CFPS system, we performed a systematic screening of auxiliary components commonly found in traditional prokaryotic CFPS protocols (Kigawa et al., 1999; Levine et al., 2019). Our objective was to identify dispensable components while maintaining or enhancing protein synthesis efficiency, thereby simplifying system preparation and reducing costs. Starting with a comprehensive reaction mixture containing up to 35 components (Kigawa et al., 1999), we iteratively evaluated the contribution of individual constituents through luciferase reporter assays. Throughout this optimization, essential core components such as creatine phosphate (CrP), creatine kinase (CrK), ATP, GTP, magnesium, and potassium were maintained in the base reaction mixture (Martin et al., 2018; Jiang et al., 2021; Bothe and Ban, 2024). Our systematic approach allowed us to precisely determine the impact of each component on overall protein synthesis yield, leading to the identification of both dispensable and critical factors.

**Figure 1.**
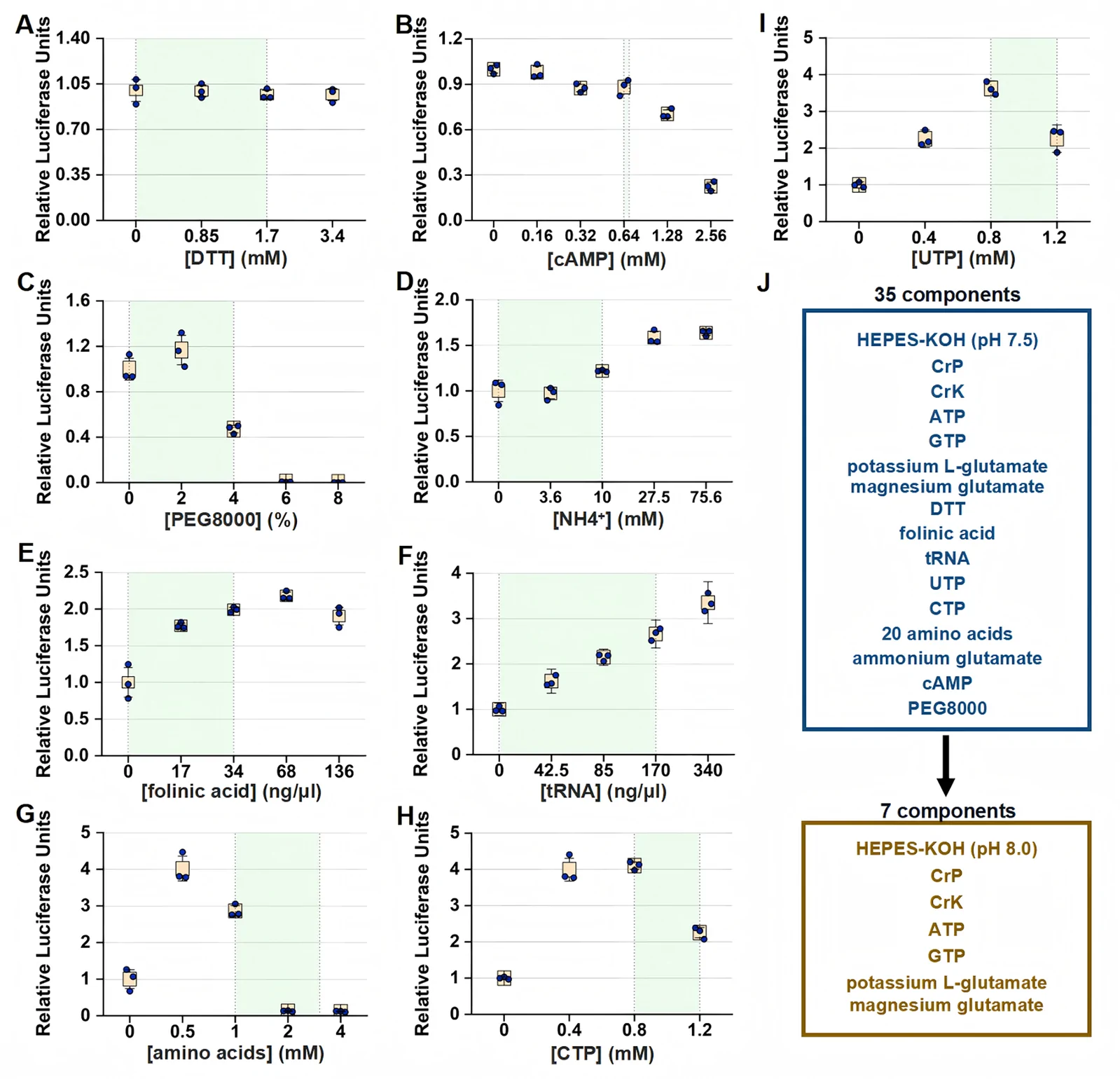
Optimization of *e*CFPS components. Protein expression levels from the *e*CFPS system were measured using a Nanoluciferase (NLuc) reporter DNA. Green area in the graphs indicate the common concentration range used in published protocols for *e*CFPS. Error bars represent the standard error (SE) of at least three independent reactions. (**A–E**) Protein expression levels of the *e*CFPS system supplemented with different concentrations of DTT (**A**), cAMP (**B**), PEG8000 (**C**), NH_4_^+^ (**D**), and folinic acid (**E**). Data present mean ± SEM, n=3 independent biological replicates. (**F–I**) Protein expression levels of *e*CFPS with various concentrations of tRNA (**F**), amino acids (**G**), CTP (**H**), and UTP (**I**). Data present mean ± SEM, n=3 independent biological replicates. (**J**) A summary of the supplement components before and after optimization. The quantitative data underlying the plots in this figure are provided in Figure 1—source data 1. Figure 1—source data 1.Plotted values in panels A—I.

Through this systematic screening, we first identified several components that could be entirely removed from the reaction mixture without compromising protein synthesis efficiency (Figure 1). Dithiothreitol (DTT), a reducing agent commonly included in both transcription and translation systems to maintain protein sulfhydryl groups in a reduced state and prevent aggregation, was found to be unnecessary within our specific system, as its removal did not affect the final protein expression levels (Figure 1A). Similarly, cyclic adenosine 3′,5′-monophosphate (cAMP), a regulator implicated in transcriptional regulation (Sands and Palmer, 2008; Daniel et al., 1998; Boshart et al., 1990), was also found to be dispensable for efficient protein synthesis (Figure 1B). Furthermore, we systematically evaluated other auxiliary components, including the molecular crowder polyethylene glycol 8000 (PEG8000), used to enhance macromolecular crowding (Chu et al., 2023; Ge et al., 2011; Liebau et al., 2024; Paudel et al., 2018; Zhang et al., 2021); ammonium ions (NH_4_^+^), typically included as an osmolyte and for its role in maintaining protein stability and solubility in *e*CFPS reactions (Zhang et al., 2021; Auton et al., 2011; Mojtabavi et al., 2019; Hunt et al., 2025; Cai et al., 2015); and folinic acid, which serves as a crucial cofactor for nucleotide synthesis (Jiang et al., 2021; Cai et al., 2015). Our results consistently showed that removing these components had a negligible impact on overall protein synthesis performance (Figure 1C–E). These findings collectively demonstrate that a substantial portion of the auxiliary components in traditional *e*CFPS protocols can be eliminated, paving the way for a more streamlined and cost-effective system.

**Figure 1—figure supplement 1.**
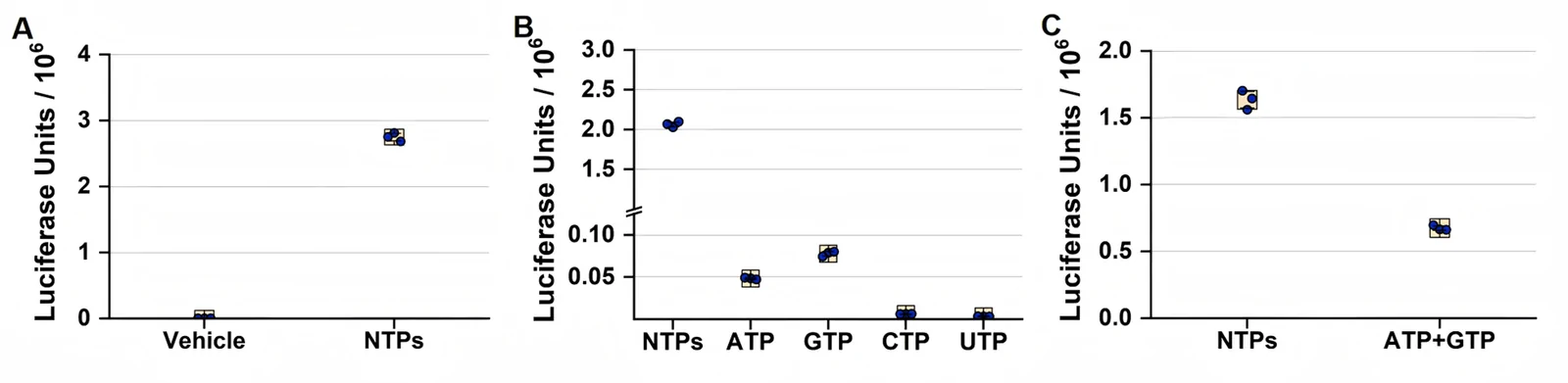
The roles of NTPs in the reaction efficiency of *e*CFPS. (**A**) Reaction efficiency of *e*CFPS supplemented with and without NTPs. (**B**) Reaction efficiency of *e*CFPS supplemented with a complete mix of NTPs or individual NTPs (ATP, GTP, CTP or UTP). (**C**) Reaction efficiency of *e*CFPS supplemented with a mix of NTPs or a combination of ATP and GTP. Data from all panels present mean ± SEM, n = 3 independent biological replicates.The quantitative data underlying the plots in this figure are provided in Figure 1—figure supplement 1—source data 1. Figure 1—figure supplement 1—source data 1.Plotted values in panels A—C.

An evaluation of various concentrations of amino acids and tRNA was conducted, as these components are fundamental building blocks for protein synthesis (Anderson, 1969; Deutscher, 1984; Rodnina, 2018). Previous studies on *e*CFPS have highlighted the rapid degradation of certain amino acids, such as arginine, cysteine, and tryptophan, necessitating their replenishment for prolonged synthesis (Kim and Swartz, 2000; Kim and Swartz, 1999). Our results showed that while these components are critical for achieving high yields, protein synthesis still occurs in their absence (Figure 1F and G). This suggests that while they are optimizable, tRNA and amino acids are not strictly essential for the reaction to proceed, likely due to residual amounts within the cell lysate (Figure 1F and G). Furthermore, we evaluated the role of nucleoside triphosphates (NTPs) in our system, which are essential for coupled transcription-translation. Interestingly, we found that protein synthesis could proceed without the addition of CTP or UTP (Figure 1H and I and Figure 1—figure supplement 1A). While adding either ATP or GTP alone resulted in a very weak reaction, the presence of both ATP and GTP together recovered the reaction to approximately 40% of the complete NTPs mix (Figure 1—figure supplement 1B and C). This highlights the critical role for ATPase-dependent chaperones (e.g., DnaK) and GTPase-dependent elongation factors (e.g., EF-Tu and EF-G; Agirrezabala and Frank, 2009), which are crucial for proper protein folding during synthesis (Mayer, 2021).

Ultimately, this comprehensive screening allowed us to successfully reduce the core reaction components from 35 to just 7 (Figure 1J).

### Optimization of essential *e*CFPS components

**Figure 2.**
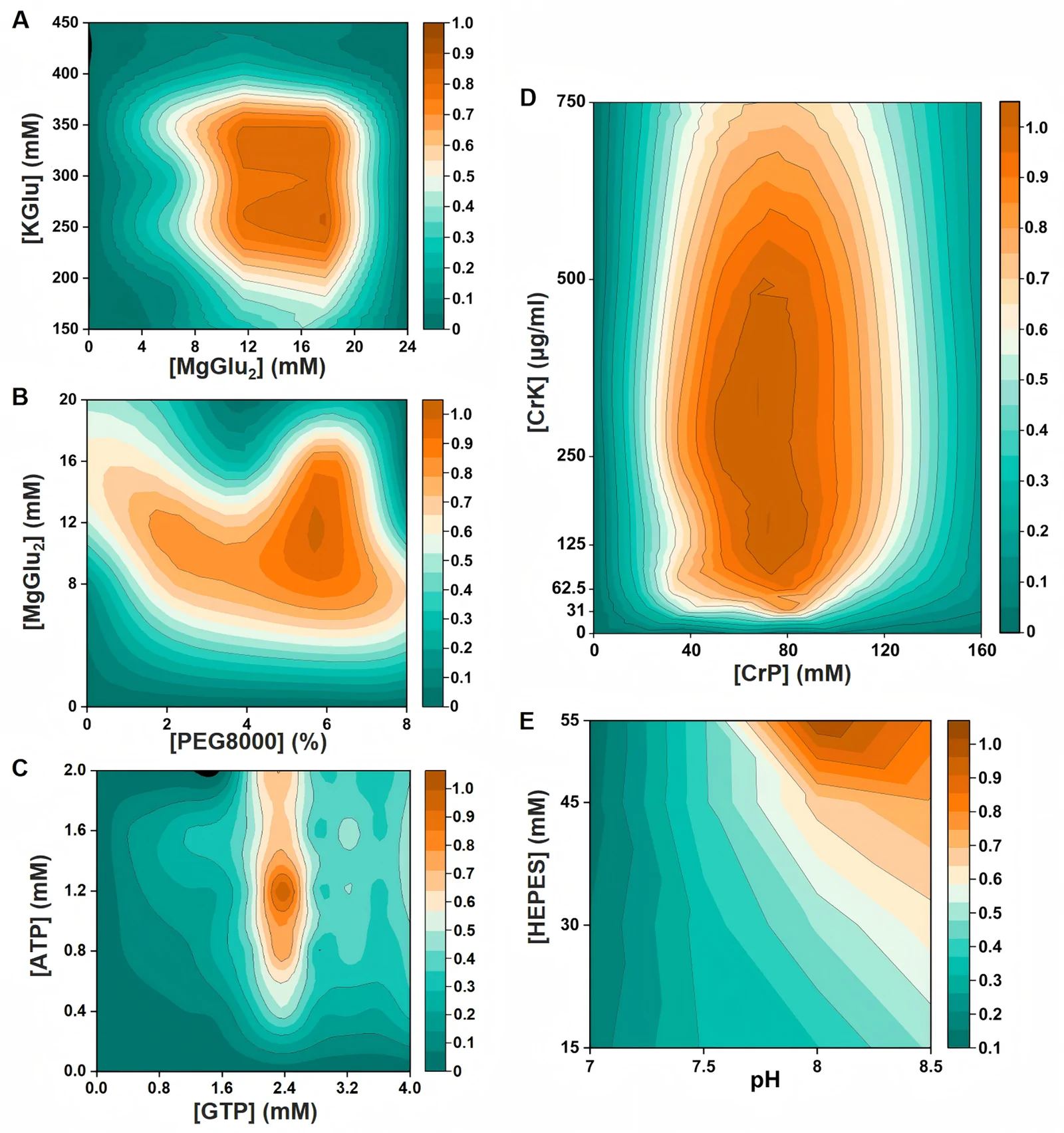
Optimization of essential components for *e*CFPS system. (**A**) Protein expression levels of the *e*CFPS system measured at varying concentrations of KGlu and MgGlu_2_. (**B**) Protein expression levels of the *e*CFPS system measured at varying concentrations of MgGlu_2_ and PEG8000. (**C**) Protein expression levels of the *e*CFPS system measured at varying concentrations of ATP and GTP. (**D**) Protein expression levels of the *e*CFPS system measured at varying concentrations of CrK and CrP. (**E**) Protein expression levels of the *e*CFPS system measured at varying pH and buffer concentrations. Data from all panels present mean ± SEM, n=3 independent biological replicates. The quantitative data underlying the plots in this figure are provided in Figure 2—source data 1. Figure 2—source data 1.Plotted values in panels A—E.

While a core set of seven components was found to be sufficient for protein synthesis, we conducted further fine-tuning to maximize the system’s performance. First, we optimized the concentrations of key salts and energy components (Figure 2). Our findings reveal that the final protein expression level is highly dependent on the concentrations of both magnesium (Mg^2+^) and potassium ions (K^+^), which are fundamental for the structural integrity and catalytic activity of ribosomes, and various enzymatic reactions critical to *e*CFPS (Nierhaus, 2014; Gesteland, 1966). Through a detailed matrix-based optimization, we first identified the optimal concentrations of Mg^2+^ and K^+^ to achieve maximum protein expression (Figure 2A). Similarly, we conducted a separate screen to optimize the concentrations of Mg^2+^ and PEG8000 as previous reports have suggested a cooperative relationship between them (Kigawa et al., 1999; Figure 2B).

**Figure 2—figure supplement 1.**
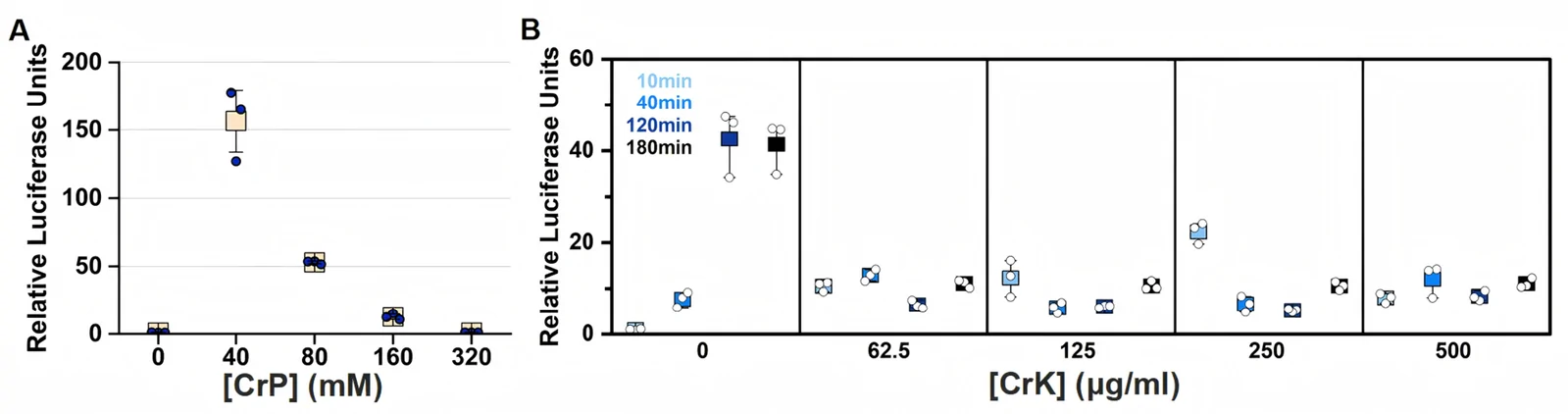
Effects of CrK and CrP on the reaction efficiency of *e*CFPS. (**A**) The reaction yield of optimized *e*CFPS at different concentrations of CrP. (**B**) Reaction efficiency of *e*CFPS at varying CrK concentrations and different reaction times. Data from all panels present mean ± SEM, n=3 independent biological replicates.The quantitative data underlying the plots in this figure are provided in Figure 2—figure supplement 1—source data 1. Figure 2—figure supplement 1—source data 1.Plotted values in panels A and B.

A comprehensive evaluation of the core energy components was also performed. We specifically focused on co-optimizing the concentrations of ATP and GTP, which serve as both energy sources and building blocks (Espinasse et al., 2020; Calhoun and Swartz, 2005; Sitaraman et al., 2004), to maximize the yield of protein synthesis (Figure 2C). Additionally, the energy regeneration system, primarily composed of CrP and CrK, is vital for maintaining sustained ATP levels (Gaddi et al., 2017; Wallimann et al., 2011). We found that CrP is crucial, with no reaction occurring in its absence (Figure 2—figure supplement 1A). Initial experiments showed that while CrK addition significantly boosted translation efficiency early on, reactions without exogenous CrK could achieve a protein expression level approximately four times higher at later time points (2 h or more), suggesting the presence of an endogenous CrK-like enzyme in the lysate (Figure 2—figure supplement 1B). A co-optimization screen for CrP and CrK concentrations was also performed, and our results showed that optimizing the concentrations of these components is critical for achieving and sustaining high protein expression (Figure 2D). Furthermore, we optimized the pH and concentration of the HEPES buffer, finding that a specific range was critical for maintaining stable and high protein expression (Figure 2E). These multifaceted optimizations ensured that each component was present at its ideal concentration, leading to a synergistic effect that significantly boosted the system’s overall performance.

### Performance characterization of the optimized *e*CFPS

**Figure 3.**
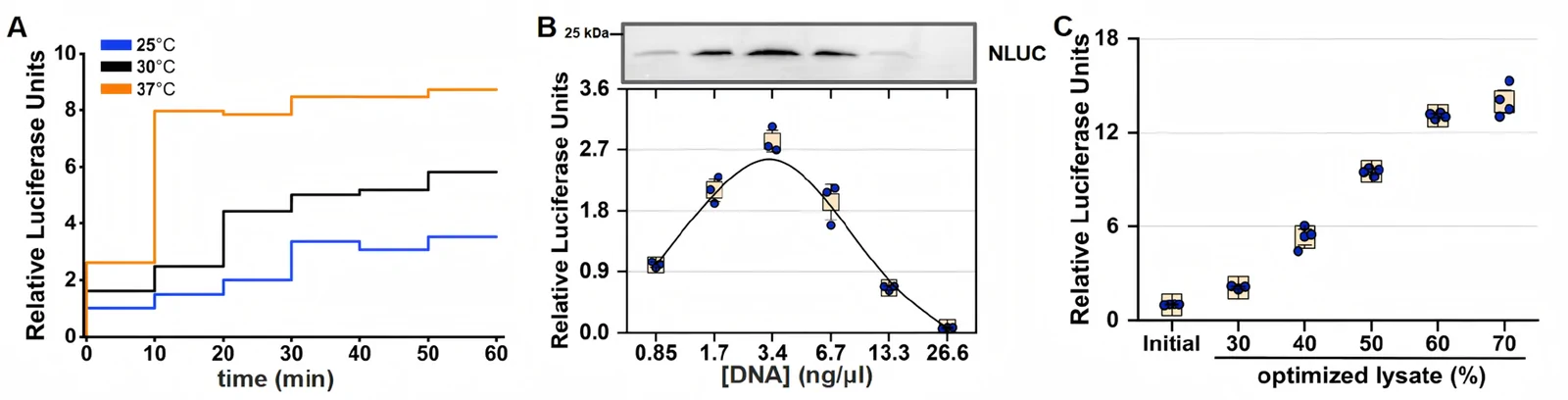
Characterization of the optimized *e*CFPS system. (**A**) Kinetics of protein synthesis at 25°C, 30°C, and 37°C over a 60-min period. Data presented as mean ± SEM; n=3 independent biological replicates. (**B**) Protein expression levels of the *e*CFPS system measured at varying DNA concentrations for a reporter encoding a FLAG-tagged NLuc. The protein product was quantified via a luminescence assay and confirmed by western blotting (upper panel). Data presented as mean ± SEM; n=3 independent biological replicates. (**C**) Comparison of protein yield. The 'initial' system denotes the traditional 35-component reaction mixture prior to optimization, serving as a baseline control for benchmarking the streamlined system. Data presented as mean ± SEM; n=4 independent biological replicates. Statistical analysis was performed using unpaired t‑test, , with each optimized group compared against the initial control. All optimized lysate groups yielded significantly higher values than the initial group (P < 0.0001). Original blots and annotated blot images corresponding to (B) are provided in Figure 3—source data 1 and Figure 3—source data 2, respectively. The quantitative data underlying the plots in this figure are provided in Figure 3—source data 3. Figure 3—source data 1.Original files for western blot analysis displayed in Figure 3B. Figure 3—source data 2.PDF files containing original western blots for Figure 3B, indicating the relevant bands. Figure 3—source data 3.Plotted values in panels A—C.

To thoroughly evaluate the performance of our optimized *e*CFPS, we conducted a series of experiments. We first investigated the kinetics of protein synthesis, measuring the expression over time at different temperatures (25°C, 30°C, and 37°C). Our results demonstrate that the system exhibits robust protein synthesis across this range, with the highest expression rates observed at the physiological temperature of 37°C (Figure 3A). Next, we assessed the system’s sensitivity to DNA concentration, finding that it could efficiently utilize a wide range of reporter DNA templates to produce quantifiable protein products, which were validated by both luminescence assays and western blot analysis (Figure 3B). Furthermore, we investigated the impact of varying cell lysate volume ratios, comparing our initial system with the newly optimized *e*CFPS. Our findings indicate that the optimized system maintains superior protein expression even at different lysate-to-buffer ratios (Figure 3C).

**Figure 3—figure supplement 1.**
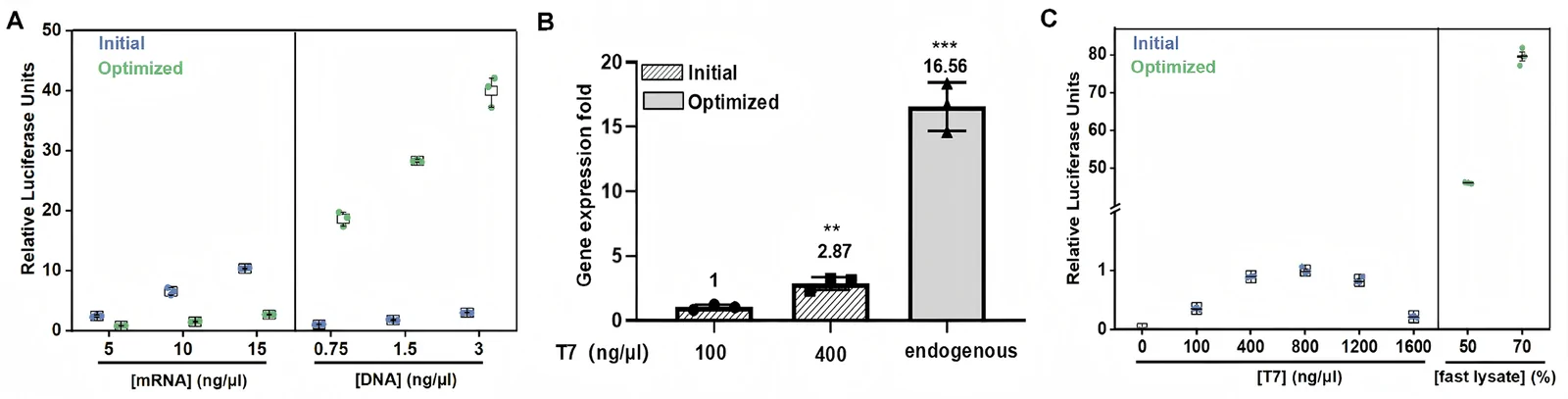
Performance evaluation and mechanistic analysis of transcriptional and translational efficiency. (**A**) Comparison between the initial and optimized systems at three different concentrations of DNA and mRNA templates to assess transcription and translation efficiency. Data are presented as mean ± SEM, n = 3 independent biological replicates. Statistical analysis was performed using unpaired t‑test. At all tested DNA template concentrations, the initial system exhibited significantly higher relative luciferase units than the optimized system (P < 0.0001). At all tested mRNA template concentrations, the optimized system exhibited significantly higher relative luciferase units than the initial system (P < 0.0001). (**B**) Quantitative RT-qPCR analysis of reporter transcript levels in the initial and optimized systems. Numbers above bars denote fold‑change relative to the 100 ng/μL T7 condition. Data present mean ± SEM, n = 3 independent biological replicates. Statistical significance is indicated: **p < 0.01, ***p < 0.001. (**C**) Comparison between the initial system supplemented with varying concentrations of T7 RNA polymerase (0–1600 ng/μL) and the optimized system. Data present mean ± SEM, n = 3 independent biological replicates. Statistical analysis was performed using unpaired t‑test. Both 50% and 70% fast‑lysate groups of the optimized system were significantly higher to all T7‑polymerase concentrations tested in the initial system (P < 0.0001). The quantitative data underlying the plots in this figure are provided in Figure 3—figure supplement 1—source data 1. Figure 3—figure supplement 1—source data 1.Plotted values in panels A—C.

To distinguish whether the increased protein expression in the optimized system was due to enhanced transcription or translation, we conducted assays using both DNA and pre-transcribed mRNA templates. The results (Figure 3—figure supplement 1A) indicate that while the translation efficiency in the streamlined system showed a slight decrease compared to the initial system, the significant improvement in transcription efficiency resulted in a higher overall protein yield. Direct quantification via RT-qPCR revealed a remarkable increase in transcript levels in our optimized system compared to the standard initial system (Figure 3—figure supplement 1B). Notably, even when the initial system was supplemented with excess T7 RNA polymerase (400 ng/µL), the accumulated transcript levels remained substantially lower than those of our optimized system. Furthermore, T7 RNA polymerase titration confirmed that the initial system saturates at 800 ng/µL—a concentration nearly 10 times higher than standard protocols (~90–100 ng/µL)—while remaining ~45-fold less productive than our optimized system (Figure 3—figure supplement 1C).

### Benchmarking and expression of challenging proteins

**Figure 4.**
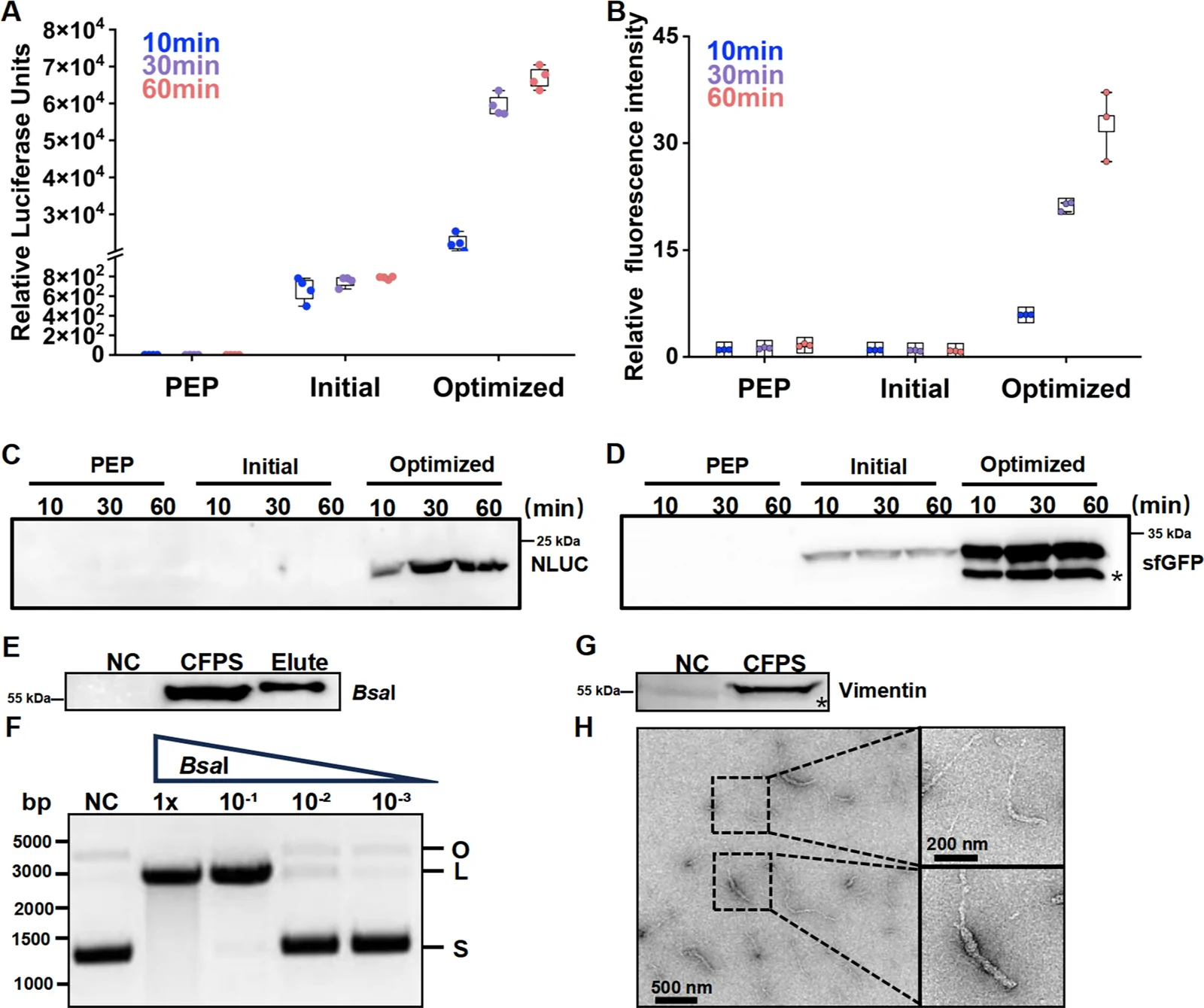
Benchmarking and expression of challenging proteins. (**A**) NLuc protein expression kinetics over time, comparing the PEP-based, initial CrP/CrK-based, and optimized CrP/CrK-based energy regeneration systems. Data present mean ± SEM, n=4 independent biological replicates. Statistical analysis was performed using unpaired t‑tests. The optimized system was significantly higher than both initial and PEP‑based groups across 10 min, 30 min and 60 min time points (P *<* 0.0001). (**B**) sfGFP protein expression kinetics over time from the three energy regeneration systems. Data present mean ± SEM, n=3 independent biological replicates. Statistical analysis was performed using unpaired t‑tests. The optimized system was significantly higher than both initial and PEP‑based groups across 10 min, 30 min and 60 min time points (P < 0.0001). (**C, D**) Western blot validation of protein expression for NLuc (**C**) and sfGFP (**D**) from the different *e*CFPS system shown in (**A, B**). Protein products were detected using an anti-FLAG antibody. The asterisk (*) indicates a non-specific band. (**E**) Western blot detection of His-FLAG-*Bsa*I expressed by the optimized *e*CFPS system using an anti-FLAG antibody. (**F**) Agarose gel electrophoresis confirming the functional activity of *e*CFPS-synthesized *Bsa*I via cleavage of a substrate plasmid. A 10-fold serial dilution of *Bsa*I was with 1x representing 0.05 mg/mL. NC (negative control) indicates no plasmid in the *e*CFPS reaction. S, L, and O indicate the respective position of the supercoiled, linear, and open circular forms of the plasmid. (**G**) Western blot analysis of vimentin expressed by the optimized *e*CFPS system using an anti-vimentin antibody. (**H**) Negative-stain electron microscopy image showing that vimentin expressed via *e*CFPS can successfully self-assemble into filaments in vitro. Scale bars: 500 nm (main panel), 200 nm (inset panels). Original blots and annotated blot images corresponding to Figure 4C/D/E/F/G are provided in Figure 4—source data 1 and Figure 4—source data 2, respectively. The quantitative data underlying the plots are provided in Figure 4—source data 3. Figure 4—source data 1.Original files for western blot analysis displayed in Figure 4C, D, E, G. Figure 4—source data 2.PDF files containing original western blots for Figure 4C, D, E, G, indicating the relevant bands. Figure 4—source data 3.Plotted values in panels A and B.

We benchmarked our system against two widely used systems: the classical phosphoenolpyruvate (PEP)-based system and our 35-component initial CrP/CrK-based system (Guzman-Chavez et al., 2022; Gregorio et al., 2019; Figure 4). We utilized NLuc, representing a broadly applicable protein, and super-folder green fluorescent protein (sfGFP), with codons optimized for *E. coli* as reporter proteins and monitored their expression over time (Figure 4A and B, Figure 4—figure supplement 1A). Our results show that the optimized *e*CFPS consistently outperforms both the classical and initial systems, achieving significantly higher protein expression levels in a shorter period (Figure 4A and B). The robust expression was further confirmed by western blot analysis, which provided clear visual evidence of the superior protein yield from our optimized system (Figure 4C and D, Figure 4—figure supplement 1B and C). Finally, we validated the function of our system through antibiotic-mediated inhibition assays. Our results demonstrate that protein expression in the optimized *e*CFPS can be effectively inhibited by standard antibiotics, confirming its robust and native-like translation machinery (Figure 4—figure supplement 1D).

**Figure 4—figure supplement 1.**
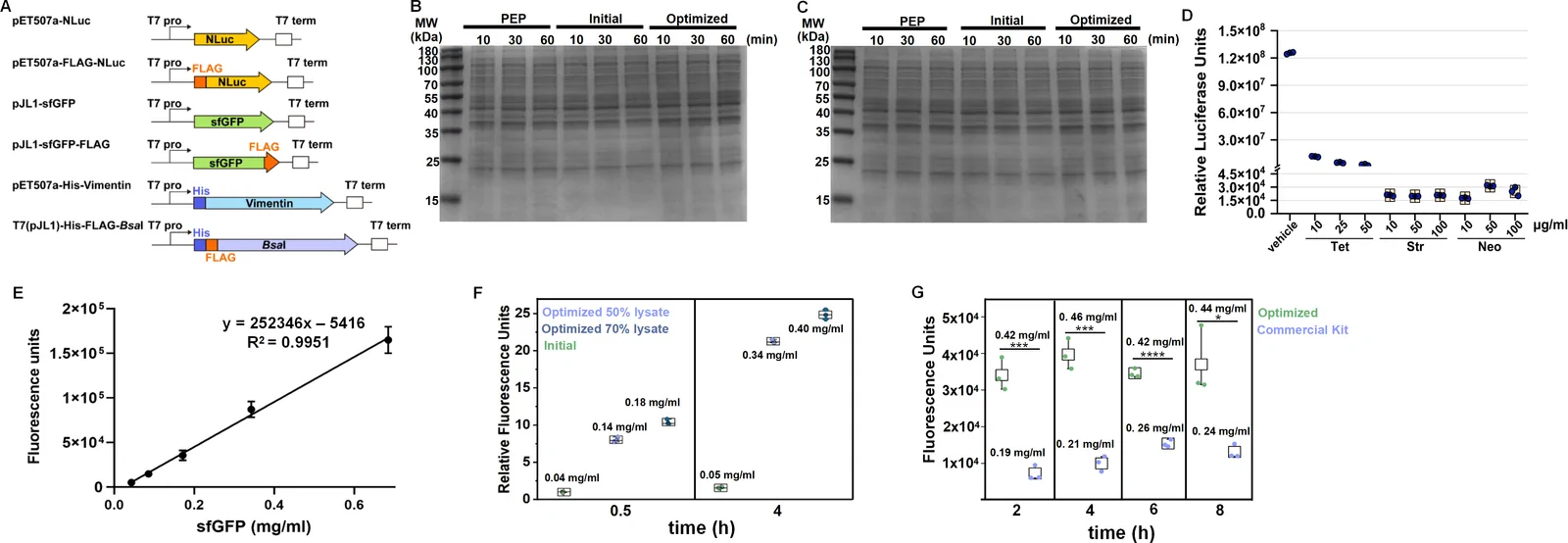
Applications of *e*CFPS. (**A**) Schematic overview of the vectors or expression cassettes used in this study, as described in the Methods section. (**B-C**) Total protein analysis by SDS-PAGE. SDS-PAGE gels for *e*CFPS reactions using NLuc (**B**) and sfGFP (**C**) as DNA templates. The SDS-PAGE results are shown as loading controls for western blot analysis of Figure 4C, D. (**D**) Validation of antibiotic-mediated reaction inhibition using the *e*CFPS system. Data present mean ± SEM, n = 3 independent biological replicates. (**E**) Standard curve correlating sfGFP fluorescence intensity with absolute protein yield. (**F**) Quantitative comparison of sfGFP protein yields between the initial and optimized systems following short (0.5 h) and long (4 h) incubation. Data present mean ± SEM, n = 3 independent biological replicates. Statistical analysis was performed using unpaired t‑tests. At both the 0.5 h and 4 h time points, the 50 % and 70 % lysate conditions of the optimized system produced significantly higher sfGFP yields than the initial system (P < 0.0001). (**G**) Benchmarking of absolute protein productivity for the optimized system against a high-end commercial cell-free system at different time points using sfGFP as a reporter. Data present mean ± SEM, n = 3 independent biological replicates. Statistical analysis was performed using unpaired t‑tests. The optimized system exhibited significantly higher sfGFP productivity relative to the commercial system at 2 h (***, P < 0.001)*,* 4 h (***, P < 0.001), 6 h (****, P < 0.0001*),* and 8 h (*, P < 0.05).Original uncropped gels and annotated gel images corresponding to Figure 4—figure supplement 1B, C are provided in Figure 4—figure supplement 1—source data 1 and 2, respectively. The quantitative data underlying the plots in this figure are provided in Figure 4—figure supplement 1—source data 3. Figure 4—figure supplement 1—source data 1.Original files for SDS‑PAGE gel displayed in Figure 4—figure supplement 1B, C. Figure 4—figure supplement 1—source data 2.PDF files containing original SDS‑PAGE gel for Figure 4—figure supplement 1B, C, indicating the relevant bands. Figure 4—figure supplement 1—source data 3.Plotted values in panels D—G.

To further validate the system’s capability in synthesizing challenging, functional proteins, we focused on the restriction endonuclease *Bsa*I (Figure 4E and F). Restriction endonucleases like *Bsa*I are notoriously difficult to express in vivo due to their specific DNA-cleaving activity, which is highly cytotoxic to the host *E. coli* cells. Traditional recombinant expression requires the compulsory co-expression of a corresponding methylase to protect the host genome, adding significant complexity to the system (Hochuli et al., 1988; Watanabe et al., 2009). Leveraging the inherent cell-free advantage of circumventing cytotoxicity, we first demonstrated the system’s utility by synthesizing the restriction endonuclease *Bsa*I (Danna and Nathans, 1971), confirming its specific enzymatic activity via a DNA cleavage assay, which underscores the system’s ability to produce complex, functional enzymes (Figure 4E and F). We confirmed that the *e*CFPS synthesized *Bsa*I was functionally active, successfully cleaving its substrate plasmid DNA (Figure 4F). Based on the standard definition of enzyme activity (1U digests 1 μg of substrate DNA in 20 μL at 37°C in 1 h), we calculated the enzyme activity of our *e*CFPS-produced *Bsa*I to be in the range of 2×10^3^ to 2×10^4^ U/mg (Figure 4F). The efficient production of this active enzyme demonstrates the power of our simplified system for producing complex, cytotoxic proteins.

Next, we successfully expressed vimentin, an intermediate filament protein (Figure 4G). Vimentin is a type III intermediate filament protein that forms part of the cytoskeleton (Eibauer et al., 2024). It is known to be difficult to express and handle due to its high propensity for aggregation. Our *e*CFPS system efficiently expressed vimentin (Figure 4G). Crucially, the vimentin expressed in our CFPS system demonstrated successful in vitro self-assembly into filaments (Figure 4H), which was confirmed by negative-stain electron microscopy, confirming the system’s robust performance even for difficult-to-express, aggregation-prone proteins (Figure 4E–H). We also benchmarked the absolute productivity of our optimized system against a high-end commercial cell-free system using sfGFP (Figure 4—figure supplement 1G). The optimized system achieved an absolute yield of 0.46 mg/mL, significantly outperforming the commercial alternative (approximately 0.21 mg/mL).

### A simplified method for bacterial lysate preparation and quality control

**Figure 5.**
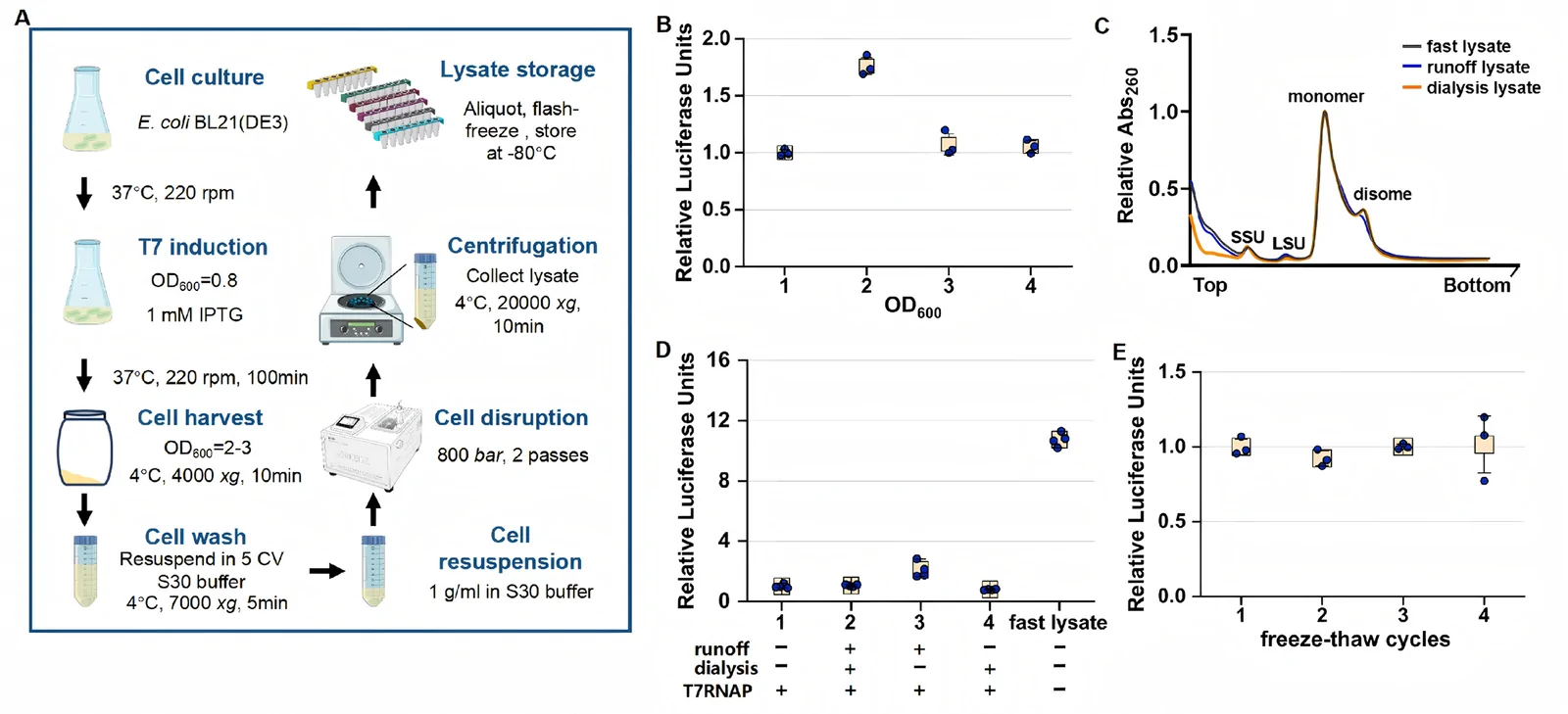
Preparation of *e*CFPS from cultured *E. coli* cells. (**A**) Flowchart of the *e*CFPS preparation procedures. (**B**) Comparison of reaction efficiency in *e*CFPS using lysate with bacteria cells harvested at different optical density. Data present mean ± SEM, n=3 independent biological replicates. (**C**) Sucrose gradient sedimentation analysis of different lysates used for *e*CFPS, revealing the presence of ribosome monomers. (**D**) Comparison of protein expression levels in *e*CFPS system using lysates prepared by runoff, dialysis, and rapid endogenous T7 RNA polymerase induction. Data present mean ± SEM, n=4 independent biological replicates. Statistical analysis was performed using unpaired t‑tests. The fast lysate (rapid endogenous T7 induction) group exhibited significantly higher units than both the runoff and dialysis groups (P < 0.0001). (**E**) Comparison of reaction efficiency in *e*CFPS using lysates after different numbers of freeze–thaw cycles. Data present mean ± SEM, n=3 independent biological replicates. The quantitative data underlying the plots are provided in Figure 5—source data 1. Figure 5—source data 1.Plotted values in panels B—E.

In addition to optimizing the reaction buffer, we sought to simplify the entire *e*CFPS procedure by developing an easy-to-use method for preparing the bacterial cell lysate. During lysate making, traditional methods, which often rely on time-consuming runoff and dialysis procedures (Levine et al., 2019; Jiang et al., 2021), were replaced with a high-pressure homogenizer for efficient cell disruption, eliminating the need for additional dialysis. Compared to traditional biochemical methods such as ultrasonication, lysozyme treatment, or freeze–thaw cycles (Fujiwara and Doi, 2016), this approach is faster and more convenient (Figure 5A). Additionally, endogenous T7 RNA polymerase in the optimized *e*CFPS lysate obviates exogenous addition, simplifying preparation and reducing costs while maintaining high translation efficiency (Figure 5A). We validated this new ‘fast lysate’ preparation by rigorous quality control. We tested lysates prepared from cells harvested at various optical densities at 600 nm (OD_600_). While the density did not significantly impact protein expression levels, a harvest optical density of OD_600_=2 yielded the best performance (Figure 5B). The quality of the lysate, particularly the integrity of its translational machinery, was further confirmed by sucrose gradient centrifugation (Figure 5—figure supplement 1A) and negative-staining transmission electron microscopy (TEM) images (Figure 5—figure supplement 1B) showing well-resolved 70S ribosomes.

**Figure 5—figure supplement 1.**
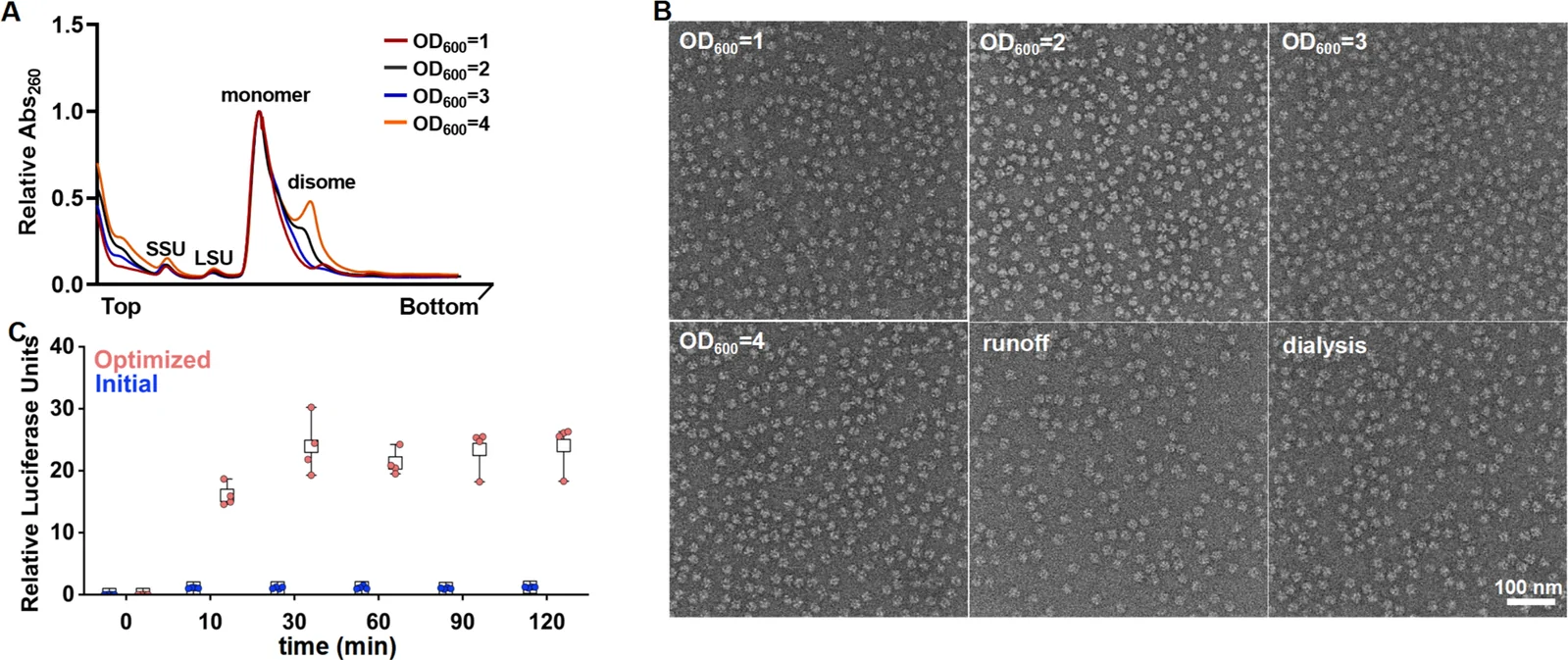
Comparison of different cell lysate preparation methods. (**A**) Sucrose gradient sedimentation analysis of cell lysates harvested at different cell densities (Optical density, was measured by absorbance at 600 nm). Peaks correspond to small ribosomal subunit (SSU), large ribosomal subunit (LSU), ribosome monomer, and disome. (**B**) Negative-staining TEM images of ribosomes isolated from different lysates. Scale bar, 100 nm. (**C**) Comparison of reaction efficiency in different *e*CFPS systems. Data present mean ± SEM, n=4 independent biological replicates. Statistical analysis was performed using unpaired t‑tests. The optimized system produced significantly higher units than the initial system across all tested time points from 10 min to 120 min (P < 0.0001). The quantitative data underlying the plots in this figure are provided in Figure 5—figure supplement 1—source data 1. Figure 5—figure supplement 1—source data 1.Plotted values in panels A and C.

To further simplify the protocol, we evaluated the necessity of the traditional runoff and dialysis steps (Jiang et al., 2021; Figure 5A). Our results from sucrose gradient analysis and TEM imaging confirmed that ribosomes remained intact, and the *e*CFPS expression signal was significantly higher without them (Figure 5C and D, Figure 5—figure supplement 1B). These findings confirm that both the runoff and dialysis steps are dispensable for our system, allowing for a significantly simplified and faster preparation. Additionally, our reliance on endogenously expressed T7 RNA polymerase yields a significantly higher expression signal, outperforming previously reported *e*CFPS systems by up to fourfold (Figure 5D).

Our optimized lysate preparation method yielded highly active and stable extracts. It is capable of sustaining protein synthesis for up to 120 min (Figure 5—figure supplement 1C) and maintains protein synthesis efficiency even after multiple freeze–thaw cycles, making it highly suitable for routine laboratory use (Figure 5E).

## Discussion

This study presents a streamlined and highly efficient *e*CFPS platform achieved by reducing the reaction components from 35 to 7. The performance of this simplified system was evaluated against the initial 35-component setup and PEP-based platforms, which serve as established reference baselines. The improvement in protein yield over these comparative standards, coupled with stable absolute productivity, supports the efficacy of the optimized reaction buffer and lysate preparation.

A notable finding was the ability of our system to function effectively without several components traditionally considered essential, which we attribute to the metabolic capacity of the fast lysate. By bypassing traditional runoff and dialysis steps, this protocol preserves active endogenous enzymes capable of synthesizing building blocks from residual precursors. For instance, functional pathways for synthesizing specific amino acids, such as deriving Cys and Trp from Ser, and generating Asn and Gln from Asp and Glu, are maintained (Yokoyama et al., 2010). Similarly, endogenous nucleotide metabolic enzymes, powered by the CrP/CrK regeneration system, convert residual pools into functional CTP and UTP to support coupled transcription and translation (Weng et al., 1986). Further analysis indicates that the performance gain is primarily driven by enhanced transcription, which more than compensates for a modest reduction in translational efficiency in the streamlined environment. This improvement does not stem from a simple increase in T7 RNA polymerase availability; instead, it reflects a systemic synergy where the streamlined buffer and the optimized metabolic environment of the lysate together alleviate transcriptional bottlenecks inherent in traditional platforms. This systemic rescue of transcription is directly supported by our RT-qPCR analysis, which confirmed that relieving buffer complexity, rather than merely increasing polymerase dosage, is key to overcoming transcriptional bottlenecks. Ultimately, this metabolic self-sufficiency, combined with the preservation of critical endogenous factors through the omission of dialysis, maintains the lysate in a highly active state that permits significant simplification of the reaction chemistry while driving superior performance.

The applicability of the optimized *e*CFPS was tested through the synthesis of targets beyond standard reporters. The production of active *Bsa*I restriction enzyme, a cytotoxic and difficult-to-express protein, and the functional assembly of vimentin, a difficult-to-handle intermediate filament protein (Eibauer et al., 2024), further validate the superior robustness and translational quality of our optimized system.

Despite its efficiency, certain limitations are acknowledged. As a prokaryotic platform, it lacks the eukaryotic chaperone systems (e.g., Hsp70/Hsp90 families) and post-translational modification (PTM) machinery required for many human proteins (Zemella et al., 2015). While we demonstrated that DTT is dispensable for the targets tested here, specialized cysteine-rich proteins may still require exogenous reducing agents to ensure proper folding. Furthermore, while the fast lysate protocol significantly lowers the barrier to entry, the expression of certain membrane proteins will likely still require the systematic screening of detergents or synthetic lipids to ensure proper integration and stability. In conclusion, our optimized *e*CFPS system reduces reaction complexity while improving performance. By simplifying both the buffer composition and the lysate preparation procedure, we have established a cost-effective and user-friendly platform. This system is expected to facilitate high-throughput protein engineering, compound screening, and diagnostic development across the broader scientific community.

## Methods

### Plasmid construction

All plasmids and primers used in this study are detailed in Supplementary file 2. The NLuc and sfGFP reporter genes, each fused with a FLAG-tag, were cloned into a T7-driven expression vector. The sfGFP gene was optimized for *E. coli* codon usage to ensure efficient translation. The vector backbone was constructed using standard molecular biology techniques.

The *Bsa*I linear DNA templates were constructed from three DNA fragments amplified by PCR. To enhance template stability and mitigate nuclease degradation within the CFPS system, approximately 300 bp sequences were incorporated upstream and downstream of the *Bsa*I coding sequence. The fragments encoding the T7 promoter/upstream sequence (Fragment 1) and the T7 terminator/downstream sequence (Fragment 2) were amplified from the pJL1 plasmid. Separately, the gene fragment for the restriction endonuclease *Bsa*I was amplified from the pUC_*Bsa*I plasmid (Fragment 3). These three fragments were subsequently fused using overlap PCR to generate the final linear template, which was confirmed to lack the recognition sequence for the target *Bsa*I enzyme.

### *E. coli* cell culture and lysate preparation

*E. coli* S30 cell lysate for CFPS was prepared using a simplified protocol adapted from a previously reported method (Jiang et al., 2021). Briefly, *E. coli* BL21(DE3) was cultured in LB medium at 37°C with shaking. Once the OD_600_ reached 0.8, endogenous T7 RNA polymerase expression was induced with 1 mM IPTG. The culture was then grown for an additional 2 h before being harvested (when the OD_600_ reached 2–3). The cells were collected by centrifugation at 4000 *g* for 10 min at 4°C. The cell pellet was washed with 5 volumes of cold S30 buffer (10 mM HEPES-KOH pH 7.5, 14 mM Mg(OAc)_2_, 60 mM KOAc) and then resuspended in S30 buffer at a ratio of 1 mL per gram of wet cell paste. Cell lysis was performed using a high-pressure homogenizer at 800 *bar* for two passes. The lysate was then clarified by centrifugation at 20,000 × *g* for 10 min at 4°C, and the resulting supernatant, designated as the ‘fast lysate’, was collected without subsequent runoff or dialysis steps. This omission ensures maximal retention of endogenous components, contributing directly to the observed system efficiency. Lysate quality control was assessed by (i) sucrose density gradient analysis and negative-staining TEM to confirm the integrity of 70S ribosomes, and (ii) functional testing by monitoring the synthesis and activity of the challenging restriction enzyme, *Bsa*I, and vimentin. Finally, the fast lysate was aliquoted and flash-frozen in liquid nitrogen before being stored at –80°C until use.

A step-by-step protocol is provided in the supplementary protocol.

### *e*CFPS reactions for luciferase reporter assay

The optimized *e*CFPS reaction was performed in a 10 μL total volume containing the components listed in Supplementary files 3 and 4. Plasmid of NLuc reporter gene was used as the template.

### Quantitative real-time PCR (RT-qPCR)

To evaluate the transcriptional levels of the NLuc reporter gene across different cell-free systems, quantitative real-time PCR (RT-qPCR) analysis was performed.

Each reaction was carried out in a total volume of 200 μL with a template plasmid concentration of 3.4 ng/μL. The reactions were incubated at 37°C for 30 min. A parallel 0 min reaction control was established as the baseline calibrator. Total RNA was extracted using the TRIzol reagent (Invitrogen) according to the manufacturer’s protocol, and the extracted RNA pellet was dissolved in an equal volume (900 μL) of nuclease-free water.

To synthesize cDNA, an equal volume (2 μL) of total RNA from each sample was reverse transcribed using the HiScript III 1st Strand cDNA Synthesis Kit (Vazyme, R312). To thoroughly rule out plasmid or genomic DNA contamination, no-template controls (NTC) and no-reverse transcriptase (no-RT) controls for all systems were prepared and analyzed in parallel. Quantitative real-time PCR was performed using the ChamQ SYBR Color qPCR Master Mix (Vazyme, Q411) on an Applied Biosystems Real-Time PCR System (Thermo Fisher Scientific) according to the manufacturer’s instructions. The relative fold changes of the NLuc transcript levels were calculated using the comparative 2^-ΔΔCt^ method, with the 0 min sample of each group serving as the calibrator. All assays were performed with three independent biological replicates.

### CFPS expression, protein purification, and analysis

Protein expression levels were quantified using NLuc and sfGFP reporter systems. NLuc activity was measured with the Nano-Glo luciferase assay system (Promega). Reaction mixtures were diluted as specified in the source data to prevent signal saturation and then analyzed on a microplate luminometer (BERTHOLD, Centro XS^3^ LB 960) to obtain the luminescence units as described previously (Li et al., 2019; Zhang et al., 2025). Relative Luciferase Units (RLU) were determined by normalizing the raw luminescence intensity of each experimental group to that of a designated control group (typically the ‘initial’ 35-component system). This normalization was performed by dividing the absolute signal of the sample by the mean signal of the control, allowing for consistency across different experimental batches and measurement sessions. sfGFP fluorescence was used to quantify protein synthesis following the methods reported (Wang et al., 2023). Briefly, 5 µL of the reaction mixture was added to 195 µL of fluorescence assay buffer (20 mM HEPES-KOH pH 7.5, 100 mM NaCl, 5 mM magnesium glutamate). Fluorescence was measured on a Cell Imaging Multimode Reader (BioTek, Cytation 5) with an excitation wavelength of 485 nm and an emission wavelength of 528 nm.

Western blotting analysis was performed to confirm protein synthesis of the *e*CFPS reactions. Synthesized proteins were detected using a primary anti-FLAG (Sigma, Cat#: M185-3L). Chemiluminescence was detected using a Gel Imaging System (Tanon).

### Preparation of a sfGFP standard curve

To establish a standard curve for the absolute quantification of sfGFP yield, *E. coli* BL21(DE3) transformed with the pJL1-sfGFP plasmid was inoculated into 5 mL of LB medium containing kanamycin and cultured overnight. Cells were harvested by centrifugation (10,000 × *g*, 5 min), resuspended in lysis buffer (50 mM Tris-HCl pH 7.5, 300 mM NaCl), and disrupted using a high-pressure homogenizer at 800 *bar* for two passes. The clarified supernatant (10,000 × *g*, 20 min) was purified via His-tag affinity chromatography, using 30 mM and 300 mM imidazole for washing and elution, respectively, with purity confirmed by SDS-PAGE.

The purified sfGFP was quantified via an Enhanced BCA Protein Assay Kit (Beyotime) and serially diluted in 50 mM HEPES buffer (pH 7.5) to concentrations ranging from 0.042 to 0.68 mg/mL. Fluorescence was measured using a Cytation 5 Multi-Mode Microplate Reader (BioTek) at an excitation of 485 nm and emission of 528 nm in flat-bottom 96-well half-area black plates. Each dilution was tested in triplicate to generate a standard curve for converting fluorescence intensity into absolute protein concentration (mg/mL).

### Expression and purification of vimentin and *Bsa*I

The gene for human vimentin (GenBank NP_003371.2) was sub-cloned into the pET507a vector for expression. *Bsa*I (4 mL reaction) and vimentin (1 mL reaction) proteins were synthesized using a CFPS system. The core reaction mixture for both proteins consisted of 16 mM magnesium glutamate, 250 mM potassium glutamate, 1.2 mM ATP, 2.4 mM GTP, 80 mM CrP, 125 μg/mL CrK, 13.3 ng/μL or 7.2 ng/μL DNA template, and 50% cell extract.

For *Bsa*I purification, the 4 mL reaction mixture was centrifuged at 20,000 × *g*, and the supernatant was incubated with 600 μL of Ni beads for 1 h at 4°C. The beads were washed five times with 2 mL of wash buffer (40 mM HEPES-KOH pH 7.5, 50 mM imidazole, 500 mM NaCl). Elution was performed with 2 mL of elution buffer (40 mM HEPES-KOH pH 7.5, 500 mM imidazole, 500 mM NaCl). The eluted *Bsa*I was desalted using a column (desalting buffer: 40 mM HEPES-KOH pH 7.5, 500 mM NaCl, 1 mM DTT), concentrated to 1 mg/mL, and stored at –80°C in the presence of 10% glycerol. *Bsa*I enzymatic activity was validated in a 20 μL reaction containing 2 μL of 10x rCutsmart (NEB), 2 μg of a substrate plasmid, and *Bsa*I enzyme diluted in a 10-fold gradient. The reaction was incubated at 37°C for 1 h and visualized via agarose gel electrophoresis.

Vimentin synthesis was carried out in a 5 mL nuclease-free tube with static incubation at 37°C for 13 h. The *Bsa*I synthesis was performed in a 10 mL nuclease-free tube at 30°C with shaking at 150 rpm for 13 h.

### Vimentin filament assembly

Vimentin, expressed via *e*CFPS, was refolded and prepared for assembly through a multi-step gradient dialysis protocol. The protein was dialyzed at room temperature in a buffer (5 mM Tris−HCl pH 8.5, 1 mM EDTA, 0.1 mM EGTA, 5 mM β-ME) sequentially containing 6 M, 4 M, and 2 M urea, with each step lasting 30 min. Subsequent overnight dialysis was performed at 4°C in the same buffer lacking urea. The protein was finally dialyzed for 1 h at room temperature into 5 mM Tris pH 8.5, 5 mM β-ME, ensuring the protein was in its tetramer configuration, after which the sample was concentrated. To initiate filament assembly, 20 μL of the concentrated protein solution was quickly mixed with 20 μL of assembly buffer (5 mM Tris pH 8.5, 170 mM NaCl, 100 mM KCl, 5 mM MgCl_2_) in a 200 μL PCR tube. The assembly reaction was incubated for 1 h in a PCR thermocycler preheated to 25°C before being immediately transferred to ice. Filament formation was subsequently verified by negative-stain transmission electron microscopy.

### Negative-staining transmission electron microscopy (TEM)

Samples from vimentin assembly, the TEM samples were prepared by negative staining. A glow-discharged, carbon-coated copper grid was first rinsed with 3 μL of protein buffer (5 mM Tris pH 8.5, 5 mM β−ME). Then, 3 μL of the assembled sample solution was incubated on the grid for 20 s. The sample was fixed using 3 μL of 0.8% glutaraldehyde solution for 20 s. Finally, the grid was stained twice with 3 μL of uranyl acetate solution, with each staining step lasting 90 s. Samples were observed using a Talos F200C transmission electron microscope at a nominal magnification of 92,000.

Samples from the SDG were prepared for TEM. Fresh, glow-discharged carbon-coated grids were immersed in 3 μL of the corresponding sample for 2 min. Excess solvent was removed, and the grids were stained with uranyl acetate for 1 min and air dried.

### Sucrose density gradient (SDG) analysis

A volume of 170–180 μL of lysate was loaded onto a 10–40% (w/w) SDG. The gradient buffer contained 10 mM HEPES-KOH (pH 7.5), 14 mM Mg(OAc)₂, 60 mM KOAc. The gradient was prepared using a simple diffusion method (Biocomp) and centrifugation at 38,000 rpm for 2.5 h at 4°C in a Beckman SW41Ti rotor. Following centrifugation, the gradient was fractionated using a density gradient fractionation system (Biocomp), with continuous monitoring of the absorbance at 260 nm.

### Statistics

GraphPad Prism v.8.0 was used to perform statistical analysis. Significance was determined by comparing two groups using the *t*-test analysis, and p<0.05 was considered statistically significant. The data are represented as the mean ± SEM. The numbers of technical replicates or biological replicates for each group are indicated in the figure legends.

## Data Availability

All data generated or analyzed during this study are included in the manuscript and the supplementary information. Specifically, source data files have been provided for all figures, and the optimized reaction components and concentrations are detailed in the main text and Supplementary Files. All plasmids and linear DNA templates used are described in the Methods section.

## Additional files

Supplementary file 1. Chemical composition of *e*CFPS buffers from previous studies (xlsx).

Supplementary file 2. Plasmid or expression cassette construct and primers (xlsx).

Supplementary file 3. Chemical composition of *e*CFPS buffers used in this study (xlsx).

Supplementary file 4. Key reagents and resources (xlsx).

MDAR checklist
