# Supplementary material for: A simplified and highly efficient cell-free protein synthesis system for prokaryotes
Source: eLife. 2026 Sep 8;14:RP109495. doi: 10.7554/eLife.109495 (PMC13553064; doi:10.7554/eLife.109495)
Supplement: Supplementary file 3 [file elife-109495-fig3-data2.zip › Figure 3-source data 2/Figure 3B relevant bands clearly labelled.pdf]

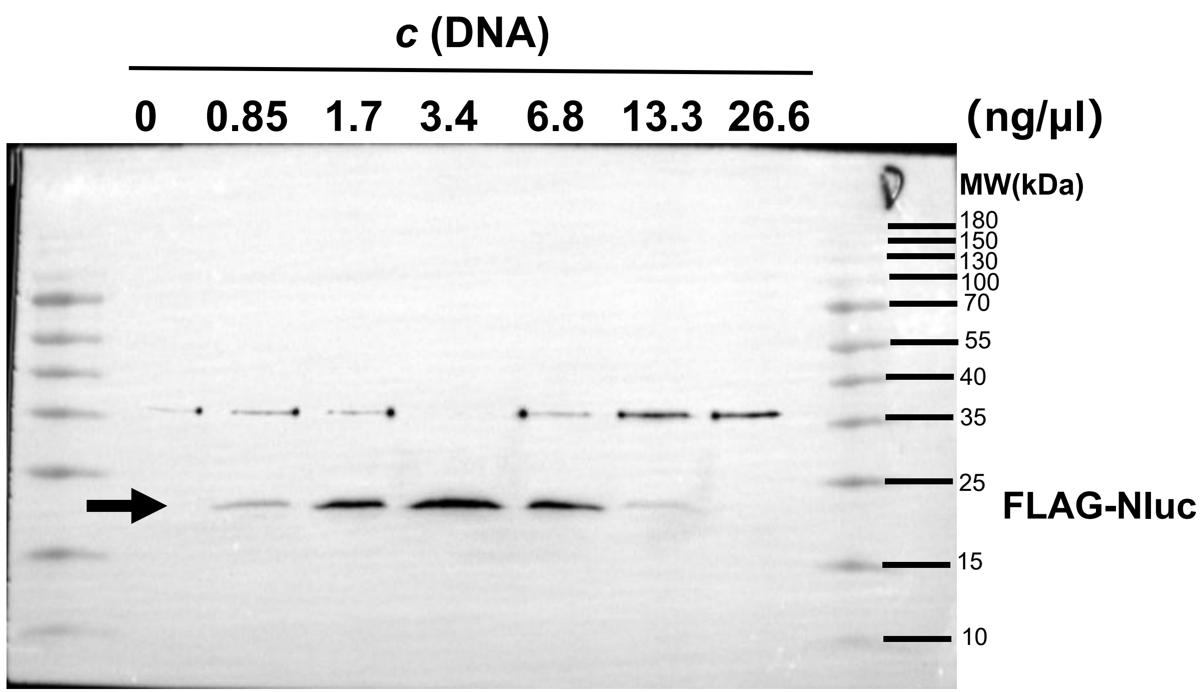

c (DNA)
0
0.85
1.7
3.4
6.8
13.3
26.6
(ng/µl)
MW(kDa)
180
150
130
100
70
55
40
35
25
15
10
FLAG-Nluc
