# Supplementary material for: A simplified and highly efficient cell-free protein synthesis system for prokaryotes
Source: eLife. 2026 Sep 8;14:RP109495. doi: 10.7554/eLife.109495 (PMC13553064; doi:10.7554/eLife.109495)
Supplement: Supplementary file 5 [file elife-109495-fig4-data2.zip › Figure 4-source data 2/Figure 4E relevant bands clearly labelled.pdf]

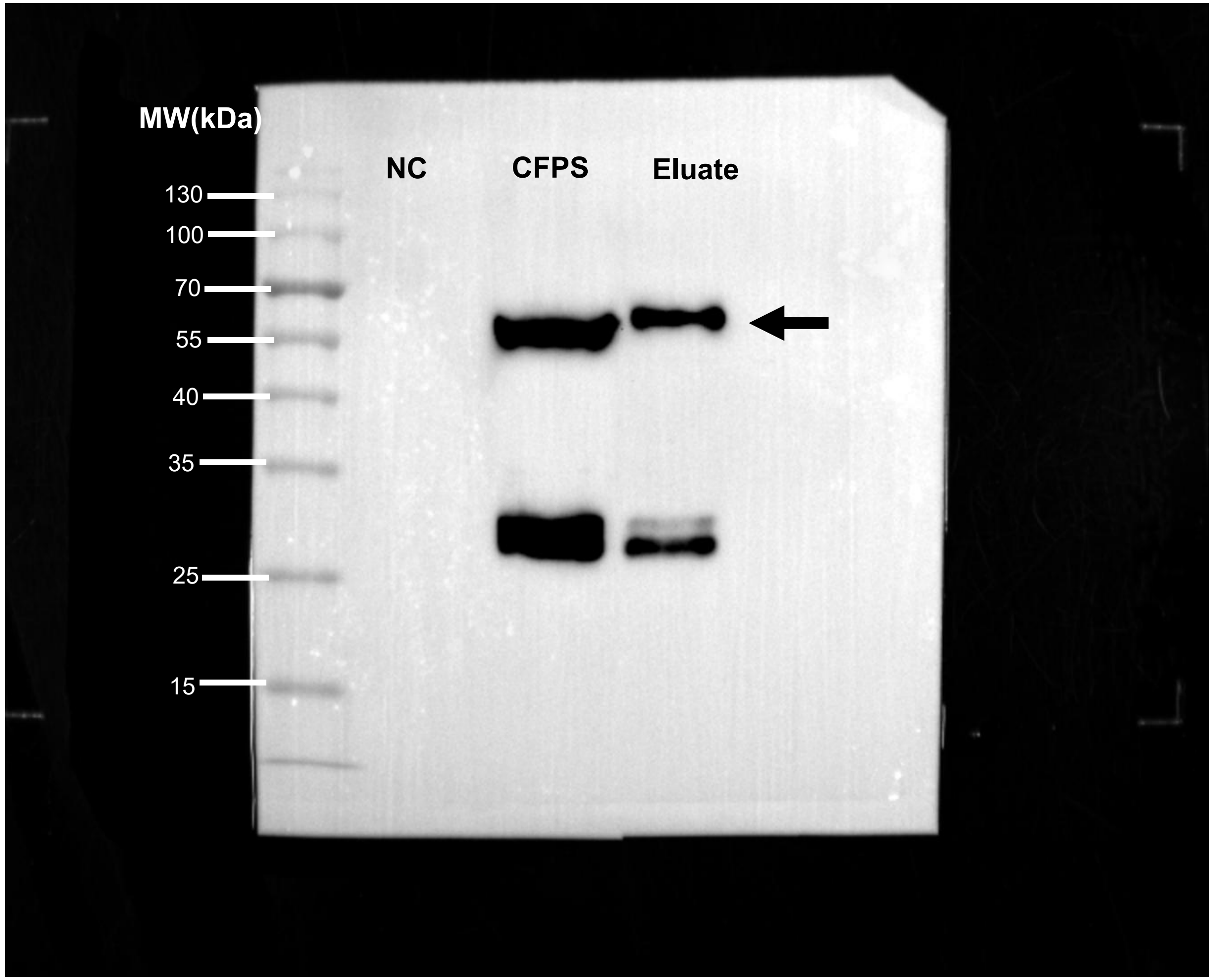
MW(kDa)
NC
CFPS
Eluate
130
100
70
55
40
35
25
15
