# Supplementary material for: A simplified and highly efficient cell-free protein synthesis system for prokaryotes
Source: eLife. 2026 Sep 8;14:RP109495. doi: 10.7554/eLife.109495 (PMC13553064; doi:10.7554/eLife.109495)
Supplement: Supplementary file 5 [file elife-109495-fig4-data2.zip › Figure 4-source data 2/Figure 4F relevant bands clearly labelled.pdf]

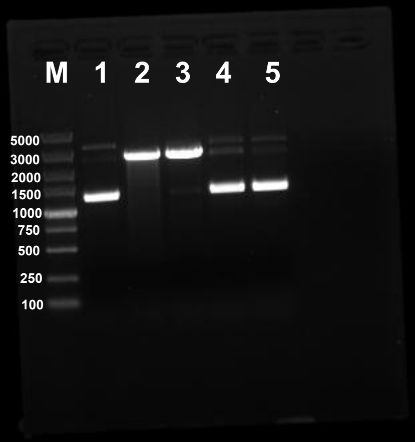

M: Marker
1: uncut pJL1 (2 μg)
2: *Bsa*I 0.5 x10^{-1} mg / ml
3: *Bsa*I 0.5 x10^{-2} mg / ml
4: *Bsa*I 0.5 x10^{-3} mg / ml
5: *Bsa*I 0.5 x10^{-4} mg / ml
