# Supplementary material for: A simplified and highly efficient cell-free protein synthesis system for prokaryotes
Source: eLife. 2026 Sep 8;14:RP109495. doi: 10.7554/eLife.109495 (PMC13553064; doi:10.7554/eLife.109495)
Supplement: Supplementary file 7 [file elife-109495-fig4-figsupp1-data2.zip › Figure 4-figure supplement 1-source data 2/Figure 4-figure supplement 1B relevant bands clearly labelled.pdf]

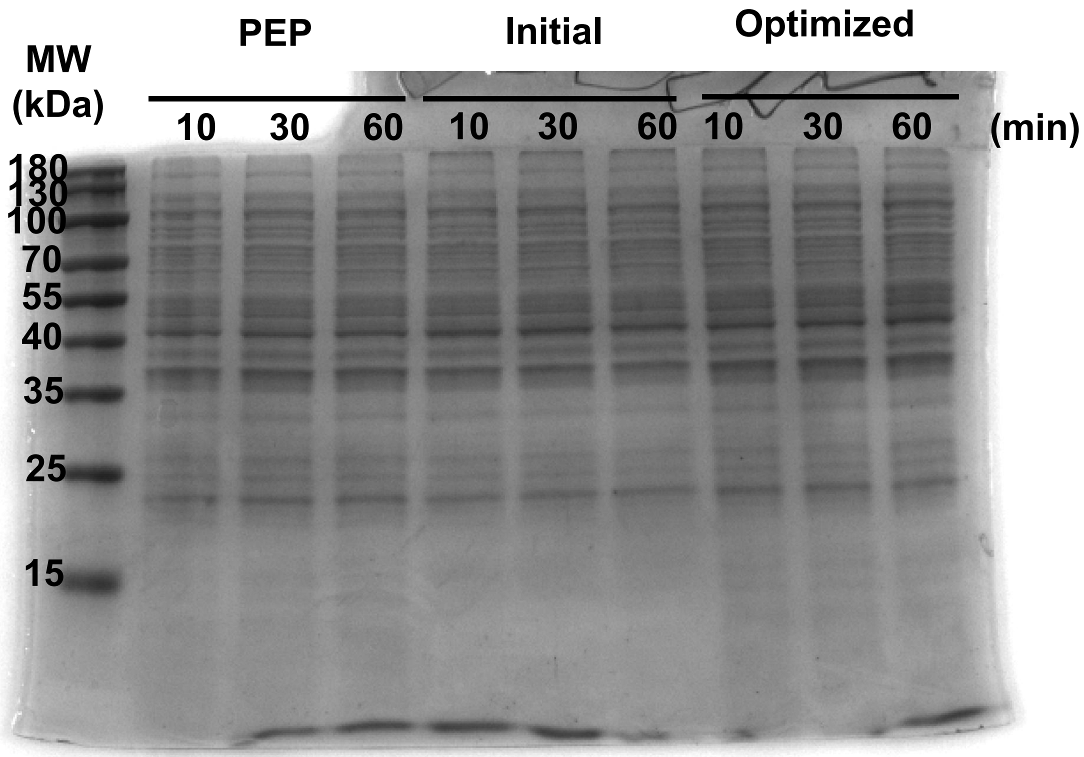
MW (kDa)
PEP
Initial
Optimized
10 30 60 10 30 60 10 30 60 (min)
180
130
100
70
55
40
35
25
15
