# Supplementary material for: A simplified and highly efficient cell-free protein synthesis system for prokaryotes
Source: eLife. 2026 Sep 8;14:RP109495. doi: 10.7554/eLife.109495 (PMC13553064; doi:10.7554/eLife.109495)
Supplement: Peer review history

# Editor report: eLife Assessment

**DOI:** 10.7554/eLife.109495.5.sa0

**Authors:** Warren Andrew Andayi

**Affiliations:** Murang'a University of Technology Kenya

**Keywords:** Convincing, Valuable

The study presents **valuable** findings of a new *E. coli* cell-free protein synthesis (eCFPS) system that has been simplified by reducing the number of core components from 35 to 7; furthermore, the findings communicate a simplified ‘fast lysate’ preparation that eliminates the need for traditional runoff and dialysis steps. The system's robustness is exhibited by its applicability to nanoluc, a ubiquitous protein and to more challenging proteins like vimentin and the active restriction endonuclease *Bsa*I. The evidence is **convincing** and supports the main claims on efficiency backed up by investigations on the mechanisms. The paper will be of interest to scientists in cell and molecular biology, microbiology, biotechnology, and protein synthesis.

# Referee report: Reviewer #1 (Public review)

**DOI:** 10.7554/eLife.109495.5.sa1

[Editors' note: this version has been assessed by the Reviewing Editor without further input from the original reviewers. The authors have addressed the comments raised in the previous round of review.]

Summary:

The authors presented a simplified *E. coli* cell-free protein synthesis (eCFPS) system reduces core reaction components from 35 to 7, improving protein expression levels. They also presented a "fast lysate" protocol that simplifies extract preparation, enhancing accessibility and robustness for diverse applications.

Strengths:

The authors present a valuable new protocol for eCFPS, which simplifies its application.

# Referee report: Reviewer #2 (Public review)

**DOI:** 10.7554/eLife.109495.5.sa2

Summary:

The authors have made a convincing argument that the current system of in vitro translation using *E. coli* extracts can be significantly optimized to work with much lesser components, while maintaining activity. They have showcased their improved activity using not only physical but also functional readouts.

Strengths:

The experiments are designed in a very logical and easy to understand manner, which makes it easier not only to follow the paper, but also reproduce the results. Functional assays with the synthesized proteins are a good way to demonstrate functionality and applicability of the system. They also benchmark their system against a commercial kit to show superior performance of their system.

Weaknesses:

The production of the lysate requires special instrumentation, limiting accessibility.

Comments on previous version:

Thank you to the authors for addressing the concerns both textually and experimentally. This work has significant value.

# Referee report: Reviewer #3 (Public review)

**DOI:** 10.7554/eLife.109495.5.sa3

Summary:

The authors aimed to overcome the challenges associated with complex, conventional prokaryotic cell-free protein synthesis (CFPS) systems, which require up to thirty-five components, by developing a streamlined and efficient *E. coli* CFPS platform to encourage broader adoption. The main objective was to reduce the number of reaction components from thirty-five to seven, while also developing an accessible 'fast lysate' preparation protocol that eliminates time-consuming runoff and dialysis steps. The authors also sought to demonstrate the robustness and translational quality of this streamlined system by efficiently synthesising challenging functional proteins, including the cytotoxic restriction endonuclease BsaI and the self-assembling intermediate filament protein vimentin.

Strengths:

This study presents several key strengths of the optimised *E. coli* cell-free protein synthesis system in terms of its design, performance and accessibility.

- The reaction mixture has been dramatically simplified, with the number of essential core components successfully reduced from up to thirty-five in conventional systems to just seven.

- The "fast lysate" protocol is a significant advance in terms of procedure.

- The system's ability to synthesise challenging, functional proteins is evidence of its robustness.

Comments on previous version.

The authors have adequately addressed my previous concerns.

# Author comment: Author response

**DOI:** 10.7554/eLife.109495.5.sa4

**Authors:** Xianshengjie Lang, Changbin Zhang, Jingxuan Lin, Zhe Zhang, Wenfei Li

**Affiliations:** Shandong University Ji'nan China; Shandong University Ji'nan China; Shandong University Ji'nan China; Shandong University Ji'nan China; Shandong University Ji'nan China

The following is the authors’ response to the previous reviews

The revisions in this version are minor and primarily include the addition of RT-qPCR validation experiments. In addition, the benchmarking data against commercial systems have now been incorporated into the main manuscript.

We also sincerely appreciate Reviewer 2 and Reviewer 3 for their highly encouraging evaluations and recognition of our system's robustness. To fully address the remaining mechanistic queries from Reviewer 1 and the benchmarking concerns from the editors, we have performed quantitative RT-qPCR to directly measure transcript levels and have integrated our commercial benchmarking data into the revised manuscript.

(1) The authors have satisfactorily addressed the concerns raised by the reviewers. However, the mechanistic basis of the observed performance gain remains insufficiently substantiated. The attribution of this improvement to enhanced transcription is currently speculative. This point could be directly tested by quantifying mRNA levels, for example, using real-time PCR, in both the initial and optimized systems. Such analysis would significantly strengthen the mechanistic interpretation of the results.

To directly validate our claims regarding transcriptional efficiency, we performed quantitative RT-qPCR to determine the transcription levels of the reporter gene in both systems.

First, we established no-reverse-transcriptase (no-RT) controls to verify complete DNA template removal. The Ct values for these controls remained above 34, confirming the absence of plasmid DNA contamination in our RNA samples.

Second, transcript levels were calculated using the comparative 2^-ΔΔCt^ method, normalized to the standard initial system (100 ng/μL T7) at 30 min. The optimized system achieved a 16.56-fold increase (P < 0.001) in transcript levels. In contrast, supplementing the initial system with high concentrations of T7 RNA polymerase (400 ng/μL) only yielded a 2.87-fold increase (P < 0.01)—which is nearly 6-fold lower than our optimized system.

These findings perfectly mirror our protein-level titration assays (Figure S3C). Supplementing the initial system with excess T7 RNA polymerase fails to rescue either transcript accumulation or protein expression. This mutual validation confirms that transcription is severely bottlenecked in traditional systems due to rapid nucleotide degradation or inhibitory reaction environments. By streamlining the reaction buffer to seven core components and omitting runoff/dialysis, our system successfully relieves these systemic bottlenecks. We have incorporated these new qPCR findings into Figure 3B, the Methods, and the Results sections of the revised manuscript.

(2) Despite the study representing an advancement towards simplifying protein expression workflows, the evidence is solid and supports the main claims however minor weakness exists i.e. the efficiency claims about the new system needs to be supported by accurate comparisons with typical cell free expression systems...

We appreciate the editor’s emphasis on establishing standard performance benchmarks. To address this important point, we would first like to highlight that our manuscript already contains extensive, rigorous benchmarking against typical cell-free platforms widely utilized in the literature. This includes detailed head-to-head comparisons with both our 35-component "initial" system and the classical, widely established "PEP-based" system across multiple expression kinetics and western blot analyses (as shown in Figure 4 and Figures S3–S4).

To fully embrace the editor's valuable recommendations regarding standard commercial performance, we are very pleased to formally integrate our commercial benchmarking data into the revised manuscript as Figure S3C.

To maintain technical neutrality, we have omitted specific brand names, presenting it generically as "a high-end commercial cell-free system." The data demonstrate that our optimized system significantly outperforms this commercial alternative in both expression speed and final absolute yield, reaching an absolute productivity of 0.46 mg/mL compared to approximately 0.21 mg/mL for the commercial kit.

We are grateful for the guidance from the editors and reviewers, which has significantly strengthened the scientific rigor of our work.
